# Operational and implementation research within Global Fund to Fight AIDS, Tuberculosis and Malaria grants: a situation analysis in six countries

**DOI:** 10.1186/s12992-017-0245-5

**Published:** 2017-03-24

**Authors:** Sabine Kiefer, Astrid M. Knoblauch, Peter Steinmann, Tanja Barth-Jaeggi, Mahnaz Vahedi, Dermot Maher, Jürg Utzinger, Kaspar Wyss

**Affiliations:** 1 Swiss Tropical and Public Health Institute, Socinstrasse 57, P.O. Box, CH-4002, Basel, Switzerland; 2 University of Basel, Basel, Switzerland; 3 The Special Programme for Research and Training in Tropical Diseases, World Health Organization, Geneva, Switzerland

**Keywords:** Implementation research, Operational research, Global Fund, Tuberculosis, Malaria, Research capacity

## Abstract

**Background:**

Operational/implementation research (OR/IR) is a key activity to improve disease control programme performance. We assessed the extent to which malaria and tuberculosis (TB) grants from the Global Fund to Fight AIDS, Tuberculosis and Malaria (“Global Fund”) include support for OR/IR, and discuss the implications of the current Global Fund operating mechanisms for OR/IR support.

**Methods:**

The situation analysis focussed on malaria and TB, while HIV was excluded. Stakeholder interviews were conducted at the Global Fund secretariat and in six purposefully selected high disease burden countries, namely the Democratic Republic of the Congo, Ethiopia, India, Indonesia, Myanmar and Zimbabwe. Interviewed in-country stakeholders included the relevant disease control programme managers, project implementation partners, representatives from international organisations with a stake in global health, academic and governmental research institutions, and other relevant individuals such as members of the country coordination mechanism. Additionally, documentation of grants and OR/IR obtained from the Global Fund was reviewed.

**Results:**

The Global Fund provides substantial resources for malaria and TB surveys, and supports OR/IR if such support is requested and the application is well justified. We observed considerable variations from one country to another and between programmes with regards to need, demand, absorption capacity and funding for OR/IR related to malaria and TB. Important determinants for the extent of such funding are the involvement of national research coordination bodies, established research agendas and priorities, human and technical research capacity, and involvement of relevant stakeholders in concept note development. Efforts to disseminate OR/IR findings were generally weak, and the Global Fund does not maintain a central OR/IR database. When faced with a need to choose between procurement of commodities for disease control and supporting research, countries tend to seek research funding from other donors. The Global Fund is expected to issue more specific guidance on the conditions under which it supports OR/IR, and to adapt administrative procedures to facilitate research.

**Conclusions:**

The importance of OR/IR for optimising disease control programmes is generally accepted but countries vary in their capacity to demand and implement studies. Countries expect guidance on OR/IR from the Global Fund. Administrative procedures specifically related to the budget planning should be modified to facilitate ad-hoc OR/IR funding. More generally, several countries expressed a need to strengthen capacity for planning, negotiating and implementing research.

**Electronic supplementary material:**

The online version of this article (doi:10.1186/s12992-017-0245-5) contains supplementary material, which is available to authorized users.

## Background

A number of proven standard interventions for controlling priority diseases in low- and middle-income countries (LMICs) exist, for example the large-scale distribution of insecticide-treated bed nets and ambulatory treatment of tuberculosis (TB) patients. At the same time, the effective implementation of these interventions requires adaptation to specific contexts, highlighting the importance of operational and implementation research (OR/IR) [1–4]. Indeed, an important aim of OR/IR is to provide an evidence base for context-specific implementation of globally proven interventions and to identify issues that prevent programmes from operating effectively, thereby providing evidence-based solutions for improving programme performance [5–8]. OR/IR intends to generate locally relevant answers and solutions that can be used by a specific programme but evidence may also be relevant far beyond [9, 10]. It is well documented that policy-making and decision-taking are not always evidence-based although local evidence is arguably the most compelling [11, 12]. It is the understanding of many global health stakeholders, including the Special Programme for Research and Training in Tropical Diseases (TDR), that OR/IR should be an integral part of disease control programme activities to maximize their outcome and impact [13–15].

The present article analyses the role that OR/IR plays for optimising programme performance of international health assistance through the example of the Global Fund to Fight AIDS, Tuberculosis and Malaria (“Global Fund”). Of note, the Global Fund is the single largest external funder of control programmes targeting TB and malaria in LMICs [16]. In 2008, the Global Fund – together with TDR – started to promote the inclusion of OR/IR activities in disease control programmes it supports. These efforts were linked to the development of a series of relevant guidelines, frameworks and toolkits [17–19]. The active promotion of OR/IR was subsequently reduced, and the extent to which countries currently request Global Fund support for OR/IR remains unclear. In 2011, the Global Fund introduced the “New Funding Model” which, among others, provides guidelines on the proven priority interventions eligible for Global Fund support. While the most recent version of these guidelines provides a detailed description of the scope of eligible interventions, hardly any reference is made to OR/IR and the role research may play for dealing with context-specific implementation problems [20]. More generally, the mechanisms determining to what degree countries take advantage of OR/IR funding are not well documented, let alone understood [1].

We conducted a situation analysis with the goal to deepen the understanding of the extent to which OR/IR activities are included in Global Fund grants and the determinants resulting in the observed patterns. Emphasis was placed on malaria and TB, while HIV was excluded. High disease burden countries in Africa and Asia receiving significant and sustained support by the Global Fund were purposefully selected to represent a range of conditions. The specific objectives of the situation analysis were: (1) to assess the extent to which Global Fund grants are being utilized to support OR/IR projects and activities; (2) to understand the context, funding sources, capacities, challenges and considerations regarding OR/IR within the selected countries; (3) to explore the priority that different stakeholders in-country and at the Global Fund give to OR/IR; and (4) to identify gaps, priorities and potentials that can be activated in the short- and medium-term.

## Methods

### Study approach

The situation analysis was conducted in the second half of 2015. A mixed methods approach was chosen, consisting of a review of key documents as well as interviews with a broad range of stakeholders. The data collection included five main activities: (i) review of publicly available documents pertaining to OR/IR and the Global Fund; (ii) review of publicly available (deadline: September 2015) grant proposals for the six selected countries; (iii) analysis of grant budgets made available to the research team by the Global Fund; (iv) interviews with representatives from the Global Fund (the fund portfolio manager (FPM) and/or respective country teams); and (v) interviews with in-country key informants from different stakeholder groups.

### Country selection

The selection of the study countries was based on the following criteria: (i) high malaria and TB burden; (ii) representation of different geographic regions; and (iii) Swiss Tropical and Public Health Institute (Swiss TPH; Basel, Switzerland) not acting as a Local Fund Agent in the country. Three countries in Africa (Democratic Republic of the Congo, Ethiopia and Zimbabwe) and three in Asia (India, Indonesia and Myanmar) were selected.

### Document review

The document review consisted of three sequential steps. First, a search on the Global Fund website was conducted to identify key documents on OR/IR and support of such activities through Global Fund grants. Second, all publicly available Global Fund proposals from Round 1 up until and including the New Funding Model that had been submitted by the six study countries were obtained and screened for evidence of OR/IR using the keywords ‘research’, ‘study’ and ‘studies’. Initially, the identified paragraphs were differentiated between ‘OR/IR’ or ‘other research’ based on the context. OR/IR-related paragraphs were classified into three categories: (1) epidemiological, behavioural and knowledge, attitude and practices (KAP) studies, and household and health facility surveys; (2) capacity building and training in OR/IR; and (3) support for the establishment of national research agendas and OR/IR review committees. Third, the available budgets were analysed with regards to the proportions spent on monitoring and evaluation (M&E) and specifically OR/IR.

### Stakeholder interviews

Interviews with key stakeholders were conducted by the authors at the Global Fund secretariat (one per country) and during visits in all study countries. At the Global Fund, the FPM and/or other country team members participated in the interview, either face-to-face or by phone. A semi-structured questionnaire guided the interview which addressed the following topics: (i) attitudes towards OR/IR and related experiences at the Global Fund; (ii) strategic perspectives on OR/IR, including funding tendencies and outlook on its relevance; (iii) barriers and challenges for successful application for, and implementing of, OR/IR; and (iv) any complementary information that might be of relevance. Answers were categorized according to pre-determined terms (for example yes/no; stakeholder type) or as suggested by interviewees (for example perceived OR/IR capacity), with explanations and answers to open questions captured in writing during the interviews. Once completed, the key points and messages of the interview were summarised and shared with the respondents for validation.

In-country, interviews were conducted face-to-face or by telephone, as appropriate. Respondents represented the following stakeholder groups: country coordinating mechanism (CCM), principal recipients and sub-recipients of Global Fund grants, national disease control programme managers, M&E and research coordinators, other project implementation partners, academic and governmental research institutions and international organizations with a stake in global health (Table 1). The following topics were addressed by the semi-structured interview guide: (i) national OR/IR policies and available funding; (ii) organisations/partners involved in OR/IR related to Global Fund-supported projects; (iii) capacities and challenges for OR/IR; (iv) dissemination of results of OR/IR and knowledge management; (v) missed opportunities and promising topics for OR/IR; and (vi) tendencies and suggestions for future OR/IR. The questionnaires are available as Additional files 1 and 2.

**Table 1:** Number of stakeholder interviews on OR/IR within Global Fund grants, stratified by respondent type and study country

Country	CCM	PR/SR/NP	Research & academia	Development partner	Total
Democratic Republic of the Congo	2	7	6	2	17
Ethiopia	1	3	4	7	15
India	1	6	2	0	9
Indonesia	1	7	3	5	16
Myanmar	0	5	1	1	7
Zimbabwe	3	4	5	6	18
Total	8	32	21	21	82

*CCM* country coordinating mechanism, *NP* national programme, *PR* principal recipient, *SR* sub-recipient

The interviews were analysed by reviewing the answers to specific topics across respondents with a focus on the detection of patterns that could be linked to respondent characteristics (for example function, represented organization type and country).

## Results

### Document review

#### Historical development

Key documents on OR/IR were typically co-authored by the Global Fund, TDR, the World Health Organization (WHO) and other organisations [17–19, 21]. Advice on the integration of OR/IR in Global Fund grants was first published in 2007. The document emphasises the importance of OR/IR for improving programme performance and outcomes and encourages applicants to earmark Global Fund resources for this purpose [17]. A guide published in 2008 further underscores the importance of OR/IR and explains relevant administrative procedures [18]. It states that “*Everyone writing a Global Fund application, and anyone concerned with improving their program’s performance, should think about whether OR should be built into the application. […] Global Fund supported programs are recommended to spend 5-10% of their grant budget on M&E, which can include spending on relevant OR”* [18]. In 2009, a framework was published for the planning and implementation of OR/IR in health and disease control programmes, including a collection of case studies [19]. OR/IR is also mentioned in the most recent M&E guidance issued by the Global Fund [21]. Taken together, these publications document a consistent and positive appreciation for OR/IR by the Global Fund. However, in the instructions and templates for the New Funding Model, no reference is made to any of the aforementioned documents and OR/IR is not explicitly featured [22, 23].

#### OR/IR in grant proposals

A total of 49 Global Fund grant proposals and concept notes were available for in-depth analysis (Table 2). Paragraphs mentioning OR/IR were identified in 92.0% of them. From the six countries analysed, most statements (54.7%) were related to epidemiological, KAP and behavioural studies and surveys of households and health facilities. Capacity building and OR/IR training was most prominent in proposals from Indonesia (21% of all relevant statements), while it featured rarely in the proposals submitted by the remaining five countries. Only a single statement concerning the establishment of national research agendas and review committees could be identified. A substantial number of references to OR/IR (34.0%) could not be classified into any of the predefined categories as they were too broad or did not mention a specific research focus or content.

**Table 2:** Number of Global Fund proposals available for review, stratified by type of statements referring to OR/IR and country

	Democratic Republic of the Congo	Ethiopia	India	Indonesia	Myanmar	Zimbabwe	Total
Reviewed proposals (n)	10	9	10	8	6	6	49
Statements on OR/IR (n)	41	29	38	22	6	26	162
Category 1 (%)	73.0	45.0	34.0	91.0	33.0	58.0	54.7
Category 2 (%)	7.0	21.0	5.0	9.0	0.0	0.0	7.9
Category 3 (%)	0.0	0.0	3.0	0.0	0.0	0.0	0.0
No specific information (%)	20.0	34.0	58.0	0.0	67.0	42.0	34.0

Category 1, epidemiological, KAP and behavioural studies and surveys of households and health facilities; Category 2, capacity building and training in OR/IR; Category 3, establishment of national research agendas and review committees

#### OR/IR and budgets

A total of 20 grant budgets from Round 6 up to the New Funding Model were available for review. Considerable variations between countries, diseases and grants were observed with regards to the allocation of funds to M&E and OR/IR. The level of detail also varied across budgets, from very general statements such as ‘*funding for OR*’ to more specific headings like ‘*conduct OR in xxx provinces to determine factors contributing to high death rate among yyy patients as well as other studies to be determined from the research agenda*’. Sometimes, the costs for different activities were detailed. According to the available budgets, between 3.5 and 39.0% of the M&E budget was dedicated to OR/IR.

### Stakeholder interviews

#### Awareness and significance of OR/IR

Key informants at the Global Fund stated that OR/IR can help to identify bottlenecks in programme implementation leading to possible solutions. The relevance of OR/IR studies to better understand programme performance and improve outcomes was less clear. The importance of large-scale and representative surveys (for example a Malaria Indicator Survey (MIS)), hence not typical OR/IR, was widely appreciated.

In-country stakeholders identified the generation of evidence on the effectiveness of an intervention and the need to enhance programme performance as main motivations to conduct research and specifically OR/IR. The majority of the respondents acknowledged the relevance of OR/IR to improve disease control programmes and were aware of the possibility to include OR/IR projects in Global Fund grant proposals.

Opinions were divided with regards to Global Fund concept note development: while some perceived the content to be country-driven, others felt it was donor-driven. The main perceived barriers to apply for OR/IR funding were: (i) limited overall funding, which leads to prioritization of curative and preventive services over OR/IR; (ii) available complementary funding for OR/IR from other sources; (iii) lack of a well-defined research agenda; (iv) limited involvement of academia during concept note development and data collection activities; (v) incompatibility of research and programme implementation, particularly the lengthy planning, approval and sometimes data collection necessary to conduct research; (vi) administrative hurdles as OR/IR needs are often vague at the time of proposal writing and later budget modifications are perceived as cumbersome; (vii) medium- to long-term time horizon for programme performance improvement through OR/IR conflicting with need for more immediate results; and (viii) interest of the Global Fund in OR/IR not clear to all stakeholders.

#### Funding of OR/IR

According to the interviewed Global Fund representatives, the inclusion of OR/IR in proposals and budgets is usually not actively promoted by the Global Fund and the relative prominence of OR/IR in different countries and proposals is mainly determined by the CCM. Key informants emphasised that countries can request funding for OR/IR studies in the frame of proposals/concept notes [21], but that the applicants are expected to take the initiative. During grant negotiations the country team of the Global Fund may try to influence the inclusion of OR/IR studies. During grant implementation, FPMs indicated that they would consider modest requests for budget reallocations to accommodate emerging needs for OR/IR but that the approval of such requests depended heavily on the proposed research and the perceived capacities in-country. This approach became entrenched since previous initiatives to streamline OR/IR handling across country teams, for example an institutional focal point for OR/IR or an inventory of all OR/IR studies funded by the Global Fund, are no longer pursued.

By 2015, the Democratic Republic of the Congo, Ethiopia and India had research agendas including OR/IR activities for both malaria and TB. Indonesia and Myanmar had identified a need for OR/IR only for the latter disease. Of note, substantial funding through a regional grant was available for malaria-related research in Myanmar (Regional Artemisinin Initiative (RAI)). Only Zimbabwe had not planned OR/IR activities pertaining to TB or malaria at the time of data collection. Stakeholders identified the Global Fund as a leading funder of large-scale surveys like Demographic and Health Surveys (DHS), MIS, and TB prevalence and mortality surveys, but small-scale OR/IR studies were often funded from other sources. The majority of the interviewees perceived the funding earmarked for OR/IR within Global Fund grants as too low considering the scarce national funding sources for research. Only in India the government allocated significant funds to research. Respondents with a history of conducting OR/IR supported by the Global Fund reported positive experiences. However, not all requests had been approved: key informants in the Democratic Republic of the Congo, Myanmar and Zimbabwe indicated that funding applications for OR/IR had been rejected in the past, and speculated that this might have been linked to perceived or real limitations in in-country capacities to implement research.

#### Implementation of OR/IR

The Global Fund representatives observed considerable diversity between countries and programmes with regards to the priority allocated to OR/IR and capacity to propose and implement OR/IR. Key informants felt that these variations could be due to (i) country priorities; (ii) implementation status of programmes; (iii) interest and influence of different stakeholder groups; (iv) available in-country capacity; (v) programme structures; (vi) bureaucratic barriers; (vii) difficulties in budgeting; and (viii) preference for funding commodities through Global Fund in the light of available alternative funding sources for OR/IR. The perception of the degree of their influence on country priorities, including the promotion of OR/IR, varied.

The main implementers of OR/IR studies funded through Global Fund grants were the Ministries of Health (MoH). Non-governmental organisations (NGOs) and academic institutions were also involved (Table 3). A main bottleneck identified by many in-country key informants was research capacity, specifically technical capacity (for example research methods), time and funding (Table 4). Technical capacity has often been reported to be concentrated within selected institutions (for example government research institutes and academic institutions), resulting in qualified staff and technical capacities being heavily centralised in the capital cities. Almost all interviewees indicated that the institutions planning or conducting OR/IR studies received technical assistance, often provided by international consultants or organisations (for example the WHO, United States Agency for International Development (USAID)/President’s Malaria Initiative (PMI), The Union Against Tuberculosis and Lung Disease (The Union), United Nations Children’s Fund (UNICEF)) but at times also by national institutions such as universities.

**Table 3:** Perceived OR/IR capacity according to in-country key informants, stratified by country

Capacity…	Democratic Republic of the Congo	Ethiopia	India	Indonesia	Myanmar	Zimbabwe	Total
…to identify OR/IR research questions (n)	10	13	7	11	6	14	61
Low (%)	30.0	8.0	0.0	27.0	33.0	21.0	19.6
Medium (%)	60.0	46.0	0.0	27.0	67.0	36.0	39.4
High (%)	10.0	46.0	100.0	45.0	0.0	43.0	41.0
…to develop study protocols (n)	12	13	7	10	6	11	59
Low (%)	42.0	15.0	0.0	20.0	17.0	9.0	18.6
Medium (%)	42.0	46.0	29.0	50.0	50.0	36.0	42.4
High (%)	17.0	38.0	71.0	30.0	33.0	55.0	39.0
…to conduct/implement OR/IR projects (n)	11	13	7	17	10	12	70
Low (%)	45.0	31.0	0.0	53.0	10.0	17.0	30.0
Medium (%)	45.0	46.0	57.0	29.0	20.0	33.0	37.0
High (%)	9.0	23.0	43.0	18.0	70.0	50.0	33.0
…to coordinate and oversee OR/IR projects (n)	12	13	7	11	6	4	53
Low (%)	50.0	15.0	0.0	64.0	33.0	0.0	32.0
Medium (%)	42.0	54.0	43.0	9.0	0.0	50.0	34.1
High (%)	8.0	31.0	57.0	27.0	67.0	50.0	33.9

**Table 4:** Research coordinating bodies, main OR/IR study implementers and key OR/IR funders, stratified by country

Country	Research coordinating body	Main implementers	Main funders
Democratic Republic of the Congo	*TB:* National TB programme *Malaria:* No official body. Stakeholders mentioned different coordination structures. Current ongoing establishment of a coordinating structure within the “Comité Scientifique”.	Ecole de Santé Publique (ESP), Department of Tropical Medicine, Centre National de Pharmacovigilance and the Institut National de Recherches Biomédicales (all associated with Kinshasa University), Swiss TPH	Global Fund, Presidents Malaria Initiative/USAID/Centers for Disease Control, Department for International Development, the World Bank, European Union, UNICEF, NGOs and foreign universities
Ethiopia	*TB:* National TB Research Advisory Committee (TRAC) *Malaria:* Malaria Research Network of Ethiopia (MRNE) The Ethiopian Public Health Institute (EPHI) has the mandate to coordinate research activities in the area of health but currently has insufficient human resources	Ethiopian Public Health Institute, local health authorities, universities	Global Fund, USAID
India	*TB:* Central TB Division, Ministry of Health and Social Welfare (MoHSW) *Malaria:* Central Malaria Division, Ministry of Health and Social Welfare; National Institute of Malaria Research; National Vector-Borne Disease Control Programme The disease control programmes maintain OR/IR committees that oversee and coordinate relevant activities	Government research institutions, Commercial research institutes	Government of India
Indonesia	*TB:* TB Operational Research Group (TORG) *Malaria:* Development of a structure similar to the TORG is ongoing. The coordination of OR for malaria is currently with the National Malaria Control Programme (NMCP)	University of Gadjah Mada, Padjadjaran University, University of Indonesia, National Institute of Health Research Development, Eijkman Institute	Global Fund, Government of Indonesia, USAID/KNCV
Myanmar	*TB:* Department for Medical Research (DMR) *Malaria:* DMR The DMR is overseeing and coordinating all research activities in the country	Department for Medical Research, University of Maryland, Mahidol University (Shoklo Malaria Research Unit), Malaria Consortium, Population Services International	Global Fund (directly and through RAI) 3 Millennium Development Goal Fund, USAID, Presidents Malaria Initiative Greater Mekong Subregion Malaria Operational Plan, WHO, Japan International Cooperation Agency
Zimbabwe	*TB:* none Malaria: none The National Institute of Health Research (former Blair Institute) has the mandate to coordinate research activities in the area of health but its current focus is on implementation of malaria research	BRTI (local NGO), universities, local health authorities, The Union, National Institute of Health Research	Global Fund, USAID

#### Coordination of OR/IR activities

In-country coordination of OR/IR was typically seen as the responsibility of either the MoH or disease-specific control programmes. Sometimes, the task had been delegated to a dedicated working group (Table 4). Important variations between countries and disease control programmes were observed with regard to governance structures. For example, in Indonesia, the Tuberculosis Operational Research Group was a well-established coordinating body but no comparable counterpart existed for malaria. In Ethiopia, both national TB and malaria programmes had established research coordination bodies; however, human resources constraints meant that their relevance was limited. In Myanmar, the coordination, execution and oversight over research is centralised in the Department for Medical Research of the Ministry of Health and Sports, while Zimbabwe lacks coordinating bodies. Results dissemination was only regulated in Myanmar and the lack of central data repositories was identified as an important weakness by the respondents.

#### Suggestions regarding OR/IR within Global Fund grants

A number of opportunities to address issues through integration of OR/IR into grants were mentioned by Global Fund representatives. However, as the organisation considers grants to be driven by country priorities, the countries and organisations with a stake in technical assistance and the development of disease-specific global guidance such as WHO and its country offices are judged to be in a better position than the Global Fund to suggest concrete OR/IR activities. The interviewed key informants agreed that earmarking a fixed percentage of the budget for OR/IR should be avoided, but that more budget flexibility would encourage countries to apply for more OR/IR studies. For instance, it was repeatedly suggested that, based on the countries research capacities, an appropriate amount should be reserved in the budget to cover emerging OR/IR needs, thus eliminating the need for formal budget re-allocations.

The main suggestions with regard to strengthening the position of OR/IR in Global Fund grants that were identified by the in-country respondents were: (i) wide dissemination of results and efforts to facilitate the uptake of research evidence by policy makers; (ii) better coordination between different institutions involved in disease control programmes and OR/IR; (iii) more inclusive approach in the concept note development process, specifically the systematic involvement of the OR/IR research community; (iv) building of research capacity; and (v) raising general awareness for the value of research. Respondents, particularly those with experience in OR/IR funding by the Global Fund, indicated that they missed specific guidance and encouragement by the Global Fund to include OR/IR activities in grants. Their suggestions to the Global Fund included: (i) provision of guidance on OR/IR inclusion; (ii) ensuring involvement of research institutions/academia in concept note development and drafting of OR/IR studies; (iii) coordination of OR/IR studies at Global Fund level to efficiently identify and address important knowledge gaps; (iv) Global Fund to request countries to outline operational and implementation challenges and suggestions to manage them; (v) support for national or disease-specific research coordination bodies to facilitate OR/IR and increase country ownership; (vi) facilitation of regional and global exchange of OR/IR findings; and (vii) alignment of funding and administration guidelines with standard research procedures.

Interviewees identified the following types of studies and topics as most important for optimizing programme implementation and grant performance: malaria and TB prevalence studies, improved service provision to migrant and hard-to-reach populations, drug resistance monitoring and mitigation strategies, malaria elimination strategies, introduction of new diagnostics, and long-lasting insecticidal nets preferences and use.

## Discussion

The Global Fund provides detailed guidance on eligible interventions under the New Funding Model, emphasises the essential role of M&E systems but does not specifically refer to the role that OR/IR may play in identifying context-specific solutions to implementation challenges. The Global Fund is a central funder with ramifications for the strengthening of health information systems and implementation of large-scale population-representative surveys pertaining to malaria, TB and HIV/AIDS [24, 25]. Basic and general research as well as individual career development grants, on the other hand, cannot be supported by the Global Fund. However, the Global Fund is committed to fund OR/IR studies that directly address operational questions and aim at identifying practical solutions to implementation bottle-necks with a view to optimize programme performance and outcomes. Exceptionally, it also supports thematic grants, which may include substantial research agendas such as the RAI that aims at interrupting the spread of artemisinin resistance in the Greater Mekong sub-region through a high coverage with long-lasting insecticidal nets, improved diagnosis and treatment, as well as strengthened surveillance.

A key finding of the current situation analysis is that there are large variations in the demand and absorption capacity for OR/IR and consequently also in available funding across the countries and programmes reviewed here. The following determinants for OR/IR related to national malaria and TB control programmes were identified: availability of a national or disease-specific research strategy, particularly one that emphasises the value of OR/IR, in-country human and technical research capacity, access to local and international technical assistance, coordination of research activities by a designated body, and involvement of all relevant stakeholders including research institution representatives in the concept note development. Of note, research needs as well as resource availability are often not well mapped out, and priorities may be poorly aligned. Similarly, a challenge appears to be the process of results dissemination, both in-country to policy makers and across countries. Last, no central database of OR/IR studies supported by the Global Fund exists, and hence, it is difficult for country teams to locate examples for proposed studies, take previous results into account, and learn from prior experience. The country teams of the Global Fund are not actively involved in concept note development as this is a country-driven process. However, interviewed in-country stakeholders expressed a wish for feedback and guidance regarding OR/IR by the Global Fund. It is conceivable that the findings from a specific country can be extended to other diseases within that country but no conclusions regarding the situation in countries not covered in this study can be made, as illustrated by the considerable variation between study countries.

Reportedly, proposing concrete OR/IR studies at the time of concept note development is often not feasible since operational challenges typically only become apparent during project implementation. While this argument is relevant for disease control programmes that are implemented over an extended period by the same in-country organisations, it might point towards an insufficient documentation of implementation challenges over the years, or inadequate involvement of relevant stakeholders in the concept note development. The results and outcome-driven funding model operated by the Global Fund means that OR/IR studies should directly contribute to improved programme performance under the same grant to be fully justified. This demand is not fully compatible with traditional research approaches under which study results are first published in academic circles and expected to be reviewed in the light of other studies to ultimately impact policy while direct feedback to relevant stakeholders is less well established.

The present situation analysis revealed that OR/IR was better integrated into grants and more prominently represented in countries with (a) an established research coordination body [26–30]; (b) a strong autochthonous capacity to design and implement research projects; (c) an inclusive approach to concept note development; and (d) a country team at the Global Fund secretariat with an active interest in integrating OR/IR studies into grants. Importantly, countries appear to often make a rational choice when confronted with the question of whether or not to include requests for support for OR/IR studies in Global Fund grant applications. Important considerations in the process are budget restrictions and the availability of alternative funding sources for OR/IR: the needs of the programme and the chances or approval are carefully assessed to maximise overall support, and as a result countries may opt to obtain a maximum of commodities and operational support through Global Fund grants, while funding for OR/IR is obtained from other sources with different funding priorities.

Our situation analysis has a number of shortcomings that are offered for consideration. First, a comparable representation of different stakeholders was sought for all countries and country teams at the Global Fund secretariat but the total number of interviews per country and the profiles of the interviewees varied slightly depending on the availability of potential respondents and the situation in-country. Second, as in all studies primarily relying on self-reported information, a certain bias due to the background and personal interest of the respondents and their awareness of the study objectives cannot be excluded. Third, the availability and detail of grant proposals and budgets varied, and their screening with a unified set of keywords may have resulted in the erroneous exclusion of relevant items if a different wording had been used. Finally, no conclusions can be drawn regarding HIV-related grants since these were excluded from the study. However, there is no indication that the Global Fund systematically applies a different OR/IR policy to HIV as opposed to malaria and TB.

## Conclusions

Demand and significance of OR/IR vary from one country to another, mainly determined by in-country capacities to coordinate and implement studies and perceived needs. In-country stakeholders express a desire for more specific guidance pertaining to OR/IR from the Global Fund and more flexible administrative procedures. Global Fund representatives generally limit themselves to react to demands from countries and focus on evidence and outcomes as main measures for success. The results of this situation analysis informed a consultation organised by TDR in December 2015 [31] that included Global Fund representatives and other interested stakeholders. Based on the discussions during the stakeholder consultation, a set of recommendations aimed at promoting OR/IR with the overarching goal to improve programme performance was developed (Table 5). In December 2016, the Global Fund published an Information Note that explicitly mentions that operations research can be included in applications to strengthen country health information systems [32].

**Table 5:** Recommendations

ᅟ	ᅟ
Global Fund secretariat	
• Global Fund to provide specific guidance on inclusion of OR/IR in concept notes and grant budgets.	
• Global Fund to ensure necessary flexibility to fund small-scale OR/IR studies identified only after grant signing, e.g. through flexible OR/IR allocation within the M&E budget.	
• (Re-)establishment of an inventory of OR/IR studies supported by the Global Fund.	
Technical partners	
• Technical partners including WHO country and regional offices to promote and actively support the inclusion of OR/IR in country health strategies, strategic development plans, guidance documents and disease-specific agendas to increase awareness and to align Global Fund concept notes including OR/IR studies with all relevant guidance.	
Countries	
• Countries to increase awareness of the importance of OR/IR within national disease control efforts and inclusion of OR/IR in concept note development. Hence, academic and research stakeholders should be closely involved in the elaboration of concept notes.	
• Countries to strengthen capacities at all levels to coordinate research, develop research agendas as well as to plan, conduct, oversee and disseminate OR/IR. Improve communication strategies to disseminate findings to relevant stakeholders to influence policy and translate findings into improved program performance.	
• Countries to improve results dissemination and uptake in-country, and – with support from funders and development partners – facilitation of results dissemination across programs and countries (e.g. regional).	

## Additional files

Additional file 1: Questionnaire for in-country stakeholders. (DOC 155 kb)

Additional file 2: Form to systematically capture responses to questionnaire for in-country stakeholders. (XLSX 28 kb)

## Abbreviations

AIDS: Acquired Immunodeficiency Syndrome
CCM: Country Coordinating Mechanism
DHS: Demographic and Health Survey
FPM: Fund Portfolio Manager
Global Fund: Global Fund to Fight AIDS, Tuberculosis and Malaria
IR: Implementation Research
KAP: Knowledge, Attitude and Practices
LMIC: Low- and Middle-Income Country
M&E: Monitoring and Evaluation
MIS: Malaria Indicator Survey
MoH: Ministry of Health
NGO: Non-Governmental Organisation
OR: Operational Research
PMI: President’s Malaria Initiative
RAI: Regional Artemisinin Initiative
Swiss TPH: Swiss Tropical and Public Health Institute
TB: Tuberculosis
TDR: Special Programme for Teaching and Training in Tropical Diseases
The Union: The Union Against Tuberculosis and Lung Disease
UNICEF: United Nations Children’s Fund
USAID: United States Agency for International Development
WHO: World Health Organization
