# Supplementary material for: Operational and implementation research within Global Fund to Fight AIDS, Tuberculosis and Malaria grants: a situation analysis in six countries
Source: Global Health. 2017 Mar 24;13:22. doi: 10.1186/s12992-017-0245-5 (PMC5366106; doi:10.1186/s12992-017-0245-5)
Supplement: Supplementary file 1 — Questionnaire for in-country stakeholders. (DOC 155 kb) [file 12992_2017_245_MOESM1_ESM.doc]

|  |
| --- |
| Programme for Research and Training in Tropical Diseases (TDR)/WHO |
| **Questionnaire**  Operational Research within Global Fund grants |

Swiss Centre for International Health

Swiss Tropical and Public Health Institute


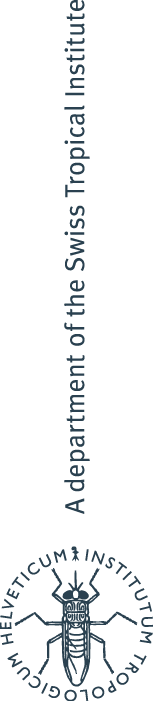


30 October 2015

Introduction and information

The Swiss TPH has been contracted by the Special Programme for Research and Training in Tropical Diseases (TDR)/WHO to conduct a situational analysis in six countries on how and to what extent countries use Global Fund (GF) grants to conduct operational research/implementation research (OR/IR). Our analysis will inform a consultation in Geneva on the 9-10th December 2015.

The review focuses on OR/IR informing malaria and tuberculosis (TB) control. OR/IR pertaining to HIV is considered to the extent that it relates to HIV/TB co-infection.

**Approach**

- The situation analysis focuses on 6 high burden countries as selected by TDR/WHO according to geographical region and disease burden (DRC, Ethiopia, India, Indonesia, Myanmar, Zimbabwe).
- The situation analysis is informed by the analysis of key documents (grant agreements, grant budgets etc.) and key informant interviews at the GF secretariat (e.g. Fund Portfolio Managers) as well as in the selected countries (Principal Recipient, Malaria/TB control programme, WHO country office, research organizations etc.).
- The interviews are analysed descriptively, with limited quantitative analysis describing the average sentiment towards specific topics. Budget analyses and OR/IR project inventories will be quantitative. Findings will be summarized in a presentation informing the consultation in Geneva, and in a report.
- The approach for the situation analysis has been developed in consultation with TDR/WHO.

**Goal of the country stakeholder interviews**

- We are interested in the in-country familiarity, experience and appreciation of OR/IR funded by the GF or benefiting GF-supported activities. This is not a full analysis of all OR/IR conducted in the country.
- Please answer the questions from your own perspective and based on your own experience. We are aware of the official documents and position of the GF regarding OR/IR.
- The interviews are confidential and the results will be anonymized in the presentation and report so that individuals cannot be identified.
- Please note that not all questions might apply to you. The questionnaire is comprehensive and will be tailored to individual stakeholders during the interview.

**In order to prepare yourself for the interview, please find attached:**

- A questionnaire, including the proposed answer categories (note: you always have the possibility to modify an answer if none of the proposed categories fits your intended meaning and we ask you to provide a brief explanation on any answer given)

Thank you already in advance for your kind collaboration.

General information

1. Name of respondent: ____________________________________

2. Organisation*:* ____________________________________

3. Date and time:

4. Country 5. Stakeholder group 6. Disease area

DRC  CCM  Malaria

Ethiopia  Principal recipient  TB

India  Sub-recipient  Malaria & TB

Indonesia  Ministry of health / national programme  TB & HIV

Myanmar  Development partner  Other: __________________

Zimbabwe  Academic / research institution

Civil society institution

Other: __________________________

A. OR/IR policies and funding

5. Does the national [TB/M] control strategy mention research (not limited to OR/IR) as an explicit key activity?

Yes

No

Don’t know

n/a

6. What motivated the inclusion or exclusion of research in the national strategy?

Need to provide evidence

Personal interests

Need to show effectiveness and efficacy

Drawing donors attention to this aspect

Aligning donor interests

Recommended from external partners

Don’t know

Other: ______________________________

Please explain your answer:

7. What motivates the program to conduct OR/IR in [TB/M]?

8. Are you aware that countries can apply for an OR/IR budget within the GF proposals?

Yes

No

Don’t know

n/a

Please explain your answer:

9. Was the inclusion of OR/IR discussed during the country dialogue resulting in the last proposal/CN submitted to the GF?

Yes

No

Don’t know

n/a

Please explain your answer:

10. Which stakeholders promoted the inclusion of OR/IR in the GF proposal/Concept Note?

PR/SR

Ministry of health / national programme

Development partner

Academic / research institution

Civil society institution

Other: __________________________

Please explain your answer:

11. Based on which arguments did you include OR/IR in the proposal/CN at the level of detail and budget as finally submitted?

12. Was the requested funding for OR/IR approved by the GF?

Yes

No

Don’t know

n/a

Please explain your answer:

B. Organizations/partners involved in OR/IR and funding

13. What are the main implementers of OR/IR projects for [TB/M] in the country?

National partners (governmental)

National partners (academic)

International partners

Other: __________________________

Please explain your answer:

14. Are the different OR/IR activities coordinated at national level?

Yes

No

Don’t know

n/a

15. If yes, how/by which body?

16. What are the key considerations when deciding which funding body to approach for support of concrete OR/IR projects?

Research is part of organisations’ core policy

Past experience in OR/IR

Active offer of the organisation to conduct OR/IR

Possibility to obtain technical support for OR/IR

Personal contacts

Donor meetings/CCM

Other: __________________________

Please explain your answer:

17. What are the main funding sources for OR/IR related to [TB/M] in your country?

GF

National sources

International sources

Don’t know

n/a

Please explain your answer:

18. Is the level of funding allocated to OR/IR for [TB/M] in the country (from your perspective) too low, too high or adequate?

Too low

Adequate

Too high

Don’t know

n/a

Please explain your answer:

19. Is the level of funding allocated to OR/IR for [TB/M] within the GF grants (from your perspective) too low, too high or adequate?

Too low

Adequate

Too high

Don’t know

n/a

Please explain your answer:

20. If there is a discrepancy between the available funding for OR/IR for malaria and TB control: Why do you think is there such a discrepancy between malaria and TB?

*This is addressed to persons that are in a position to oversee both diseases in the country.*

C. Capacities and challenges for OR/IR

21. What are the main operational challenges to conduct OR/IR in the country?

22. How to you judge the in-country capacity to identify OR/IR research questions?

Low

Medium

High

Don’t know

n/a

Please explain your answer:

23. How to you judge the in-country capacity to develop study protocols?

Low

Medium

High

Don’t know

n/a

Please explain your answer:

24. How to you judge the in-country capacity to conduct/implement OR/IR projects?

Low

Medium

High

Don’t know

n/a

Please explain your answer:

25. How to you judge the in-country capacity to coordinate and oversee OR/IR projects?

Low

Medium

High

Don’t know

n/a

Please explain your answer:

26. Is obtaining ethical approval for OR/IR a challenge?

Yes

Somewhat

No

Don’t know

n/a

Please explain your answer:

27. Do you receive technical assistance (TA) to conduct OR/IR projects?

Yes, in-country

Yes, international

No

Don’t know

n/a

Please explain your answer:

28. Which organizations do provide TA for OR/IR projects or promote OR/IR in general?

D. Results of OR/IR and knowledge management

29. Are you aware of an example how the results of OR/IR modified a program design or implementation (e.g. improved quality, effectiveness, coverage, timeliness of interventions)?

30. Is there a policy governing OR/IR results dissemination?

Yes

No

Don’t know

n/a

Please explain your answer:

31. Are OR/IR results actively shared with other institutions potentially interested in the findings?

Yes

No

Don’t know

n/a

Please explain your answer:

32. With which stakeholders are the OR/IR results regularly shared?

PR/SR

National programme

GF (Geneva)

Public

Other: ________________________

n/a

Please explain your answer:

33. In which format are OR/IR results normally shared?

Reports

Scientific publications

Data sets

Presentations, posters

Policy briefs

Webpages

Other: ________________________

n/a

Please explain your answer:

E. Missed opportunities and quick wins

34. What are the key topics/research questions where increased OR/IR could play an essential role in optimising grant/programme implementation?

*Examples*

35. Which policy and operational adjustments to the current procedures related to OR/IR would result in the biggest programme improvement?

F. Tendencies and suggestions

37. Over time, do you observe a change within the country in terms of the recognition for the importance of OR/IR?

Yes

No

Don’t know

n/a

Please explain your answer:

38. Over time, do you observe a change in terms of the amount of funding for OR/IR through GF grants?

Yes

No

Don’t know

n/a

Please explain your answer:

39. Over time, do you observe a change in terms of the attitude of the GF secretariat towards OR/IR?

Yes

No

Don’t know

n/a

Please explain your answer:

40. Are you able to share any lessons learned that could be relevant for other stakeholders or other countries?

41. Is there any other issue/experience/suggestion you want to share with regard to OR/IR in GF grants?
